# Global Hypomethylation as Minimal Residual Disease (MRD) Biomarker in Esophageal and Esophagogastric Junction Adenocarcinoma

**DOI:** 10.3390/cancers17162668

**Published:** 2025-08-15

**Authors:** Elisa Boldrin, Maria Assunta Piano, Alice Volpato, Rita Alfieri, Monica Franco, Tiziana Morbin, Annalisa Masier, Stefano Realdon, Genny Mattara, Giovanna Magni, Antonio Rosato, Pierluigi Pilati, Alberto Fantin, Matteo Curtarello

**Affiliations:** 1 Immunology and Molecular Oncology Diagnostics Unit, Veneto Institute of Oncology IOV-IRCCS, 35128 Padua, Italy; mariaassunta.piano@iov.veneto.it (M.A.P.); antonio.rosato@iov.veneto.it (A.R.); matteo.curtarello@iov.veneto.it (M.C.); 2 Anatomy and Pathological Histology Unit, Veneto Institute of Oncology IOV-IRCCS, 35128 Padua, Italy; alice.volpato@iov.veneto.it; 3 General Gastric and Esophagus Surgery Unit, IRCCS Humanitas Research Hospital, 20089 Milan, Italy; rita.alfieri@humanitas.it; 4 Gastroenterology Unit, Veneto Institute of Oncology IOV-IRCCS, 35128 Padua, Italy; monica.franco@aulss2.veneto.it (M.F.); tiziana.morbin@iov.veneto.it (T.M.); annalisa.masier@aulss2.veneto.it (A.M.); alberto.fantin@iov.veneto.it (A.F.); 5 Oncological Gastroenterology, Centro di Riferimento Oncologico di Aviano (CRO) IRCCS, 33081 Aviano, Italy; stefano.realdon@cro.it; 6 Surgical Oncology of Digestive Tract Unit, Veneto Institute of Oncology IOV-IRCCS, 35128 Padua, Italy; genny.mattara@iov.veneto.it (G.M.); pierluigi.pilati@iov.veneto.it (P.P.); 7 Clinical Research Unit, Veneto Institute of Oncology IOV-IRCCS, 35128 Padua, Italy; giovanna.magni@iov.veneto.it; 8 Department of Surgery Oncology and Gastroenterology, University of Padova, 35122 Padua, Italy

**Keywords:** esophageal adenocarcinoma (EADC), esophagogastric junction adenocarcinoma (EGJA), Barrett’s esophagus (BE), global hypomethylation, liquid biopsy, minimal residual disease (MRD), Methylation-Sensitive Restriction Enzyme droplet digital PCR (MSRE-ddPCR)

## Abstract

**Background/Objectives**: Esophageal and esophagogastric junction adenocarcinoma (EADC-EGJA), which mainly develops from Barrett’s esophagus (BE), low-grade dysplasia (LGD), and high-grade dysplasia (HGD), has a poor prognosis and several unmet clinical needs, among which is the detection of minimal residual disease (MRD) after endoscopic/surgical resection. Long interspersed nuclear element-1 (LINE-1), a surrogate marker of global methylation, is considered an emerging biomarker for MRD monitoring. The aim of this study was to determine, by LINE-1 methylation analysis, at which carcinogenesis step global methylation is affected and whether this biomarker could be followed in longitudinal to monitor the disease behavior post-surgery. **Methods**: Cell-free DNA of 90 patients with non-dysplastic Barrett’s esophagus (NDBE), HGD/early EADC-EGJA, or locally advanced/advanced EADC-EGJA were analyzed for LINE-1 methylation, by Methylation-Sensitive Restriction Enzyme droplet digital PCR (MSRE-ddPCR). Twenty-six patients were longitudinally studied by repetitive blood sampling. **Results**: Global hypomethylation increased during carcinogenesis, with significant difference between locally advanced/advanced EADC-EGJA and NDBE patients (*p* = 0.028). Longitudinal cases confirmed the rareness of hypomethylation in NDBE cases. The majority of HGD/early EADC-EGJA and locally advanced/advanced EADC-EGJA patients showed methylation changes after resection according to clinical status. **Conclusions**: This study suggests that global hypomethylation occurs just prior to cancer invasiveness and that it is a promising biomarker to monitor MRD.

## Simple Summary

Growing evidence supports the hypothesis that, besides genetic alterations, epigenetic events, in particular aberrant methylation, can also contribute to tumor development and malignant progression that may lead Barrett’s esophagus to evolve into dysplasia and finally into esophageal and esophagogastric junction adenocarcinoma. Moreover, a high relapse frequency after surgery among patients with locally advanced esophageal and esophagogastric junction adenocarcinoma has been observed, suggesting a need to find new biomarkers able to predict the behavior of the disease. Due to these reasons, this research aims to clarify how and when global methylation level is affected during the carcinogenesis process from Barrett’s to adenocarcinoma and if this biomarker could predict relapse. Finding such a biomarker could be of great interest; indeed, it has the potential utility to enable early intervention or adjustment to treatment strategies.

## 1. Introduction

Esophageal cancer (EC) is the eighth most commonly diagnosed cancer and the sixth most common cause of cancer death in the world [1]. In Europe, in 2020, EC accounted for about 52,993 new diagnoses and 45,551 deaths [2]. Although its burden varies greatly across countries and populations, due to differences in the prevalence of underlying risk factors, survival from EC remains low, in the range of 10–30% at 5 years post diagnosis in most countries [1].

Esophageal adenocarcinoma (EADC), the most frequent histotype of EC, occurs preferentially in the distal part of the esophagus, next to the junction with the stomach. The eighth edition of American Joint Committee on Cancer (AJCC) includes also the esophagogastric junction adenocarcinoma (EGJA) as EC when the tumor involves the junction and its epicenter is within the proximal 2 cm of the cardia [3].

Age, gender (male), smoking habit, persistent gastro-esophageal reflux disease (GERD), and obesity are all risk factors for EADC development [4,5,6].

Patients with early disease are treated with upfront surgery or endoscopic treatment, whereas in locally advanced disease, preoperative treatment based on chemoradiotherapy containing platinum, taxanes, or fluoropyrimidines is mandatory [7,8,9].

At the histological level, it is recognized that non-dysplastic Barrett’s esophagus (NDBE) is a risk condition to develop EADC and EGJA; indeed, the majority of EADC and EGJA cases arise in an area of NDBE [10].

NDBE is characterized by the substitution of normal esophageal epithelium with columnar epithelium. One of the major causes of this transformation is attributed to inflammation and cell proliferation induced by the chronic exposure of lower esophagus to acid and bile salts typical of GERD [11]. Indeed, NDBE prevalence is higher in the population with GERD (7.7%) compared with the population without this condition (<5%) [12].

NDBE can evolve to low-grade dysplasia (LGD), high-grade dysplasia (HGD), and finally invasive EADC [13]. The risk of progression of NDBE to EADC is around 0.3% per year in absence of dysplasia, but the risk rises to 5–20% in the presence of HGD [14,15]. These observations led to the activation of surveillance protocols based on repetitive endoscopies together with targeted biopsies of the suspected areas followed by random biopsy sampling of the entire NDBE segment [11].

Frequency of surveillance endoscopy is determined by the detection of dysplasia. However, the efficacy of current surveillance protocols is still a matter of debate, and there is a great need to find biomarkers that could help to identify those NDBE patients at risk of developing EADC in order to better tailor their follow-up [11].

At the molecular level, NDBE evolves to invasive EADC via the accumulation of mutations and somatic copy number alterations in tumor suppressor genes and oncogenes and the occurrence of genome-wide events such as chromothripsis, repeated breakage–fusion–bridge cycles. and whole-genome doubling [16,17].

Growing evidence supports the hypothesis that, besides genetic alterations, epigenetic events can also contribute to the acquisition of cancer hallmark capabilities during tumor development and malignant progression [18]. Among the possible epigenetic events, alteration of methylation level, primarily in the form of tumor suppressor gene hypermethylation, has been frequently found in EADC and NDBE [19].

Genome-wide methylation analysis conducted in a variety of cancers has revealed that, next to a selective hypermethylation at the CpG islands of specific tumor suppressor gene promoters, the dominant epigenetic change is global hypomethylation [20]. Long interspersed nuclear element-1 (LINE-1), since it constitutes a consistent part of the human genome (17%), has been indicated as a possible surrogate marker of global methylation [21,22]. LINE-1 analysis has been conducted in many of the most common lethal cancers, and its hypomethylation has been often associated with a poor outcome [23,24,25,26,27,28,29,30,31,32,33,34,35,36].

In EC, LINE-1 status has been principally investigated in esophageal squamous cell carcinoma (ESCC), the other main EC histotype, in which its hypomethylation is frequent and associated with poorer survival [37,38]. In EADC there are less data; however, we have previously demonstrated that LINE-1 is frequently hypomethylated both in solid and liquid biopsies of EADC patients compared to the constitutive genomic DNA isolated from peripheral blood mononuclear cells (PBMCs) [39]. The occurrence of LINE-1 hypomethylation in EADC is suggested by the demonstration that the retrotransposition of this repetitive element is active and contributes to EADC genomic instability through insertions in the coding sequence of several genes (*ERBB4*, *CTNNA3*, *CTNNA2*, *CDH18*, and *SOX5*) [40,41,42,43]. Indeed, LINE-1 retrotransposition in cancer cells seems to be associated with its aberrant hypomethylation during carcinogenesis, while, in physiological conditions, it is inhibited by its hypermethylation [44].

Due to the promising results in EADC and the demonstration of the feasibility to detect LINE-1 methylation status also in cell free DNA (cfDNA) in a comparable way to solid biopsies [39], in this work, with the aim to determine at which carcinogenesis step methylation level is affected, liquid biopsy samples from a cohort of 30 locally advanced/advanced EADC-EGJA, 30 HGD/early EADC-EGJA, and 30 NDBE patients have been analyzed for methylation of the LINE-1 biomarker.

Moreover, since it is well known that more than a half of locally advanced EADC-EGJA patients relapse after surgery [45], with a consequent emerging clinical need for the identification of minimal residual disease (MRD), in this study, a longitudinal monitoring of patients through repetitive cfDNA sampling post-surgery has been included.

Indeed, a successful detection of MRD, defined as the persistence of residual cancer cells undetectable with conventional radiological and clinical exams, could help clinicians to optimize post-operative patients’ management [46,47,48].

## 2. Materials and Methods

### 2.1. Patients

In this prospective study, a total of 90 patients, 30 diagnosed with NDBE, 30 with HGD or early EADC-EGJA, and 30 with locally advanced/advanced EADC-EGJA, have been included.

NDBE patients were defined as BE patients with a clinical history of stable disease (SD) for at least 3 years (range: 3–12 years; median: 5 years). For NDBE patients, BE lesion was defined as short or long based on its length (<3 cm or ≥3 cm), according to guidelines [11].

All the enrolled patients were recruited from the Gastroenterology Unit and/or from the Surgical Oncology of Digestive Tract Unit of Veneto Institute of Oncology IOV—IRCCS (Padua, Italy) between July 2014 and June 2023. Inclusion criteria were (i) age > 18 years and (ii) a histological diagnosis of BE, HGD/early EADC-EGJA, or locally advanced/advanced EADC-EGJA. Concurrent diagnosis of a synchronous or metachronous tumor within 5 years was an exclusion criterion. For each patient, a blood sample was collected at the enrollment for NDBE, at the diagnosis or just prior to radiofrequency ablation (RFA) or mucosectomy (MS) or surgery resection for HGD/early EADC-EGJA, and at the diagnosis or just prior to surgery for locally advanced/advanced EADC-EGJA patients.

Twenty-six of the ninety patients were also longitudinally studied by collecting serial blood samples (at least two) at the enrollment (timepoint 0) and at each follow-up visits. Longitudinally studied patients included 11 NDBE, 7 HDG/early EADC-EGJA, and 8 locally advanced/advanced EADC-EGJA patients.

Blood samples of 20 healthy volunteers (median age: 30.5 years; range: 21–65 years; males: 8, females: 12) were also included.

### 2.2. DNA Extraction

Peripheral blood samples were collected in cell-free DNA BCT tubes (Streck, La Vista, NE, USA). Plasma was isolated as described in [49]. One aliquot of whole blood was also stored for germline DNA (gDNA) extraction. cfDNA was extracted from 1 mL of plasma using a Maxwell RSC cfDNA Plasma Kit (Promega, Milan, Italy). gDNA was isolated from 500 μL of peripheral blood with a Maxwell RSC Whole Blood DNA Kit (Promega). cfDNA and gDNA quantity were assessed with a Qubit dsDNA HS Assay kit (Thermo Fisher Scientific, Monza, Italy). Quality of randomly selected cfDNA samples was evaluated by an Agilent TapeStation 4200 using a Cell-free DNA Screen Tape Assay kit (Agilent Technologies, Milan, Italy).

### 2.3. Methylation-Sensitive Restriction Enzyme Droplet Digital PCR (MSRE-ddPCR)

LINE-1 methylation was analyzed with an in-house designed assay by MSRE-ddPCR, a particular type of ddPCR that permits to discriminate methylated and unmethylated cytosines within CpG islands. ddPCR, by partitioning the sample in thousands of individual PCR reactions, offers the advantage of direct and accurate quantification of template, maximizing the chance to detect rare genetic alterations. gDNA and cfDNA of each patient were digested or not digested with HpaII (New England BioLabs, Ipswich, MA, USA), a methylation-sensitive enzyme that cuts CCGG recognition site when both cytosines are unmethylated and, with a lower efficiency, when the external cytosine is methylated (mCCGG), while it is inhibited when both cytosines are methylated (mCmCGG).

Digested and undigested DNA were amplified by ddPCR with primers for the amplification of a sequence between nucleotides 12–128 of LINE-1 promoter (GenBank accession number X58075.1). This region contains two CCGG sites.

Two different ddPCR reaction mixes of 20 µL with or without HpaII were prepared. Reaction mixes contained 10 μL of 2× ddPCR SuperMix for Probes (No dUTP) (Bio-Rad, Milan, Italy), 1 μL of 20× target LINE-1 primers/probe (FAM), 1 µL (10 U) of HpaII (for digested sample), or 1 µL of H_2_O (for undigested sample).

Pair of primer/probe was in a final concentration of 900 nM/250 nM. The primer sequences were 5′-CAAGATGGCCGAATAGGAAC (FW) and 5′-TGGCACTCCCTAGTGAGATG (RV). A DNA input of 0.01 ng/well was used as template. Each ddPCR included, as positive and negative controls, Human WGA Methylated DNA and Human WGA Non-methylated DNA (Zymo Research, Irvine, CA, USA). A no-template control was included. Droplets were generated by a QX200 droplet generator (Bio-Rad). An Applied Biosystems VeritiDx thermal cycler was used to perform the enzymatic digestion reaction at 37 °C for 2 h and, subsequently, the enzyme inactivation at 95 °C for 20′, followed by PCR amplification using the cycling conditions: 95 °C for 10′, followed by 50 cycles at 94 °C for 30″, 60 °C for 1′, and 98 °C for 10′. Each reaction was performed in two replicates.

Droplets were read with QX200 droplet reader and analyzed with QuantaSoft™ version 1.7.4 (Bio-Rad). Positive droplets, containing amplification products, were discriminated from negative ones by applying a fluorescence amplitude threshold that was set manually, as suggested by the manufacturer. The software quantified the number of copies/μL for each well as output. The mean of two replicates was calculated to obtain a more accurate value. Samples with <10,000 droplets per 20 μL of PCR reaction were excluded from the analysis.

The methylation level of the cfDNA sample of a patient, normalized to its respective lymphocyte-derived gDNA, was calculated by the following formula:

$$cfDNA\;methylation\left(\%\right)=\frac{n{}^{\circ}\;copies\;of\;digested\;cfDNA/\mu L/n{}^{\circ}\;copies\;of\;undigested\;cfDNA/\mu L}{n{}^{\circ}\;copies\;of\;digested\;gDNA/\mu L/n{}^{\circ}\;copies\;of\;undigested\;gDNA/\mu L}\times 100$$

The cfDNA methylation percentual is referred to as “LINE-1 normalized methylation” throughout this paper and figures. Raw methylation percentages of the cfDNA and the gDNA for each individual are reported separately in Table S1.

To set up the cut-off value to consider a cfDNA sample as hypomethylated, we carried out the same procedure as the patients’ samples by analyzing the cfDNA and gDNA of 20 healthy volunteers. The established cut-off value, under which the patients’ cfDNA sample was considered hypomethylated, was determined as the mean of LINE-1 normalized methylation -2SD (98.67% -2SD = 93.06%).

### 2.4. Statistics

Differences in distribution between groups for categorical variables were evaluated using the chi-squared or Fisher’s exact test. Differences between groups for continuous variables were evaluated using the Mann–Whitney or Kruskal–Wallis test. Correlation analysis was performed using Spearman’s test. Overall survival (OS) was defined as the time between the date of tumor resection and the date of death for any cause or the date of the end of the study (January 2024). Progression-free survival (PFS) was calculated as the time from the date of tumor resection to the date of progression, or the date of the end of the study. OS and PFS curves were defined using the Kaplan–Meier function and differences between strata were estimated using a log-rank test. Tests with *p*-values < 0.05 were considered statistically significant. Statistical analyses were performed using SigmaPlot version 14.0 (Systat Software Inc., San José, CA, USA). Graphs were generated using GraphPad Prism software (version 9.2 for Windows, San Diego, CA, USA).

## 3. Results

### 3.1. Clinicopathological Characteristics of Patients

The clinicopathological characteristics of NDBE, HGD/early EADC-EGJA, and locally advanced/advanced EADC-EGJA patients are reported in Table 1.

The median age was lower in NDBE patients compared with HGD/early EADC-EGJA (*p* < 0.001) and locally advanced/advanced EADC-EGJA (*p* = 0.006), while in these two latter groups it was similar (*p* = 0.425) The male/female ratio was similar in the three cohorts, with a prevalence of males, according to GLOBOCAN 2020 [1]. The majority of NDBE cases were long BE with the lesion located at the esophagus. HGD/early EADC-EGJA and locally advanced/advanced EADC-EGJA patients had the lesion/tumor located in the esophagus (EADC) or in the esophageal junction (EGJA). cStage and pStage or ypStage were reported according to Rice et al. [3].

For the HGD/early EADC-EGJA group, only early EADC-EGJA patients had a pStage because they underwent surgery, while a pStage of HGD is lacking due to endoscopic removal of the lesion by MS or RFA.

Only locally advanced/advanced EADC-EGJA patients received neoadjuvant treatment (based on a combination of platinum, taxanes, or fluoropyrimidines and radiotherapy) as recommended by guidelines [7,8,9] in almost all cases (only one patient was not treated due to treatment refusal) and received adjuvant therapy in case of pathological residual disease and suitability to receive the therapy.

### 3.2. LINE-1 Normalized Methylation Analysis in Cell-Free DNA at Baseline

**Figure 1:**
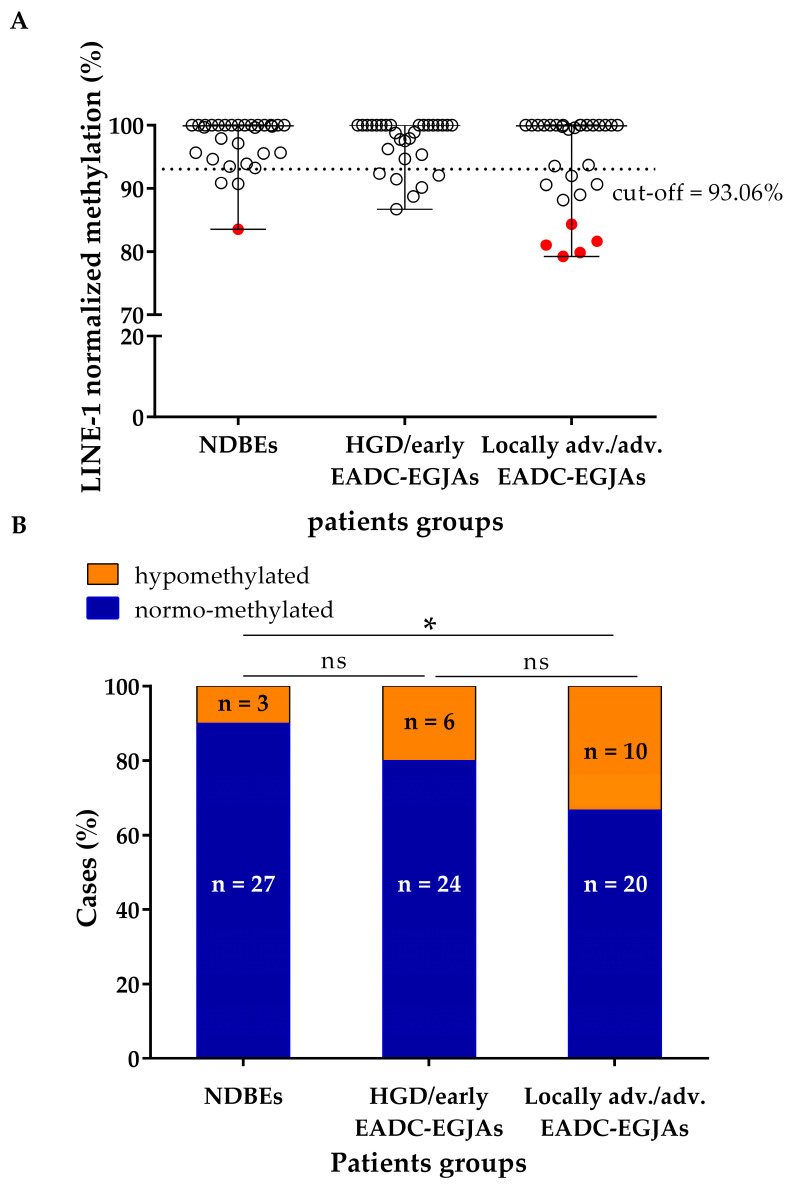
LINE-1 normalized methylation status at baseline in cfDNA samples of NDBE, HGD, and locally advanced/advanced EADC-EGJA patients. (**A**) Dot plot of LINE-1 methylation level for each group. The cut-off of 93.06%, below which the sample was considered as hypomethylated, is shown as a dashed line. Highly hypomethylated cases are shown in red. (**B**) Distribution of hypomethylated and normo-methylated cases for each group. LINE-1: long interspersed nuclear element-1; NDBE: non-dysplastic Barrett’s esophagus, HGD: high-grade dysplasia; EADC-EGJA: esophageal adenocarcinoma–esophageal junction adenocarcinoma; ns: not significant; * *p* < 0.05.

LINE-1 normalized methylation level was analyzed by MSRE-ddPCR in all patients. A cfDNA sample was considered hypomethylated when its residual methylation was below the cut-off level of 93.06%, calculated by analyzing the cfDNA samples of 20 healthy volunteers as controls. The age of the controls (median: 30.5; min: 22; max: 65) was lower compared to that of the patients (*p* < 0.0001). The distribution of the methylation level of the cfDNA samples in the three groups of patients is shown in Figure 1A.

In total, 3 out of 30 (10%), 6 out of 30 (20%), and 10 out of 30 (33.3%) cfDNA samples were hypomethylated in the NDBE, HGD/early EADC-EGJA, and locally advanced/advanced EADC-EGJA groups, respectively (Figure 1B). The locally advanced/advanced EADC-EGJA group had a significantly higher number of hypomethylated samples compared to the NDBE group (*p* = 0.028; Figure 1B), whereas the difference in the frequency of hypomethylated cfDNA samples was not significant between the NDBE and HGD/early EADC-EGJA groups and between the HGD/early EADC-EGJA and locally advanced/advanced EADC-EGJA groups (Figure 1B).

In the locally advanced/advanced EADC-EGJA group, among the hypomethylated cases, a small cluster of five “highly hypomethylated cases” ranging from 79.24% to 84.34% was distinguishable. A single isolated highly hypomethylated case was present also in the NDBE group (Figure 1A).

Considering all the hypomethylated samples of the three groups together, the overall decrease of methylation level ranged from 7.6% to 20.8%, with a median of 11% (IQR = 5.9%).

**Figure 2:**
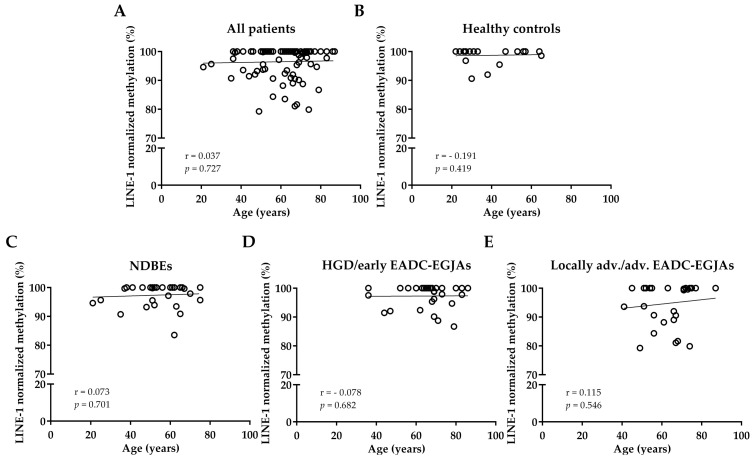
Correlation between LINE-1 normalized methylation and age in cfDNA samples of (**A**) all patients considered together; (**B**) healthy controls; (**C**) NDBE patients; (**D**) HGD/early EADC-EGJA patients; and (**E**) locally advanced/advanced EADC-EGJA patients. LINE-1: long interspersed nuclear element-1; NDBE: non-dysplastic Barrett’s esophagus; HGD: high-grade dysplasia; EADC-EGJA: esophageal adenocarcinoma–esophageal junction adenocarcinoma.

The association between LINE-1 methylation level and the clinicopathological characteristics of patients was investigated. A correlation between LINE-1 methylation values and age in all patients considered together or divided by the three groups was not observed (r = 0.037, *p* = 0.727 for all patients; r = 0.073, *p* = 0.701 for NDBE; r = −0.078, *p* = 0.682 for HGD/early EADC-EGJA; and r = 0.115, *p* = 0.546 for locally advanced/advanced EADC-EGJA patients). No correlation was observed for healthy controls either (r = −0.191, *p* = 0.419) (Figure 2).

**Figure 3:**
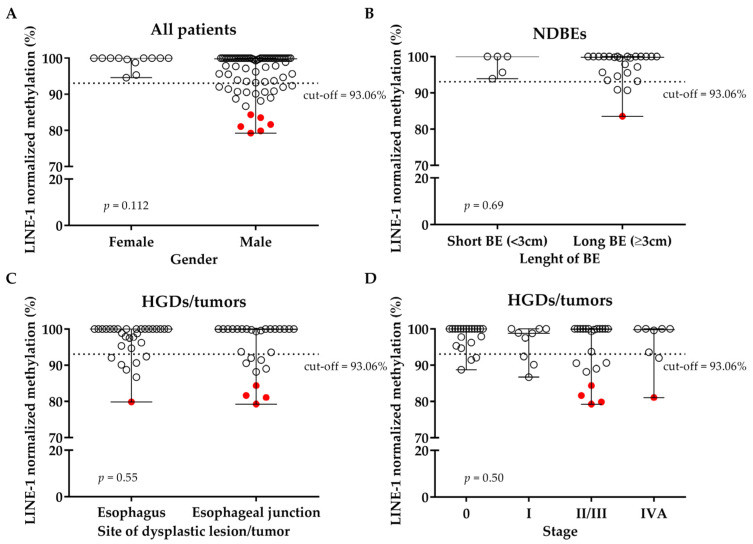
Dot plot of LINE-1 normalized methylation level according to (**A**) gender, considering all patients together; (**B**) length of BE in NDBE patients; (**C**) site of the dysplastic lesion/tumor; and (**D**) stage in the HGD/tumors. Highly hypomethylated cases are shown in red. LINE-1: long interspersed nuclear element-1; NDBE: non-dysplastic Barrett’s esophagus; BE: Barrett’s esophagus; HGD: high-grade dysplasia.

Association between methylation level and gender has been not observed (*p* = 0.112); however, all the highly hypomethylated cases were males (Figure 3A).

Concerning the NDBE group, an association with the length of BE has not been found (*p* = 0.69; Figure 3B). Moreover, in the two groups of neoplastic lesions (HGD/tumors), associations with the main clinicopathological characteristics in terms of site of dysplastic lesion/tumor and stage of the tumor at the moment of blood draw have not been found (*p* = 0.55; *p* = 0.50; respectively; Figure 3C,D). The stage at the moment of blood draw was considered cStage or p/ypStage depending on if blood draw had been collected at diagnosis or at surgery.

Interestingly, almost all the highly hypomethylated cases distinguishable in Figure 1A belonged to esophageal junction tumors with II/III-IV stages, as shown in Figure 3C,D. The only highly hypomethylated NDBE case had no apparent difference in clinicopathological characteristics compared to other NDBE cases.

**Figure A1:**
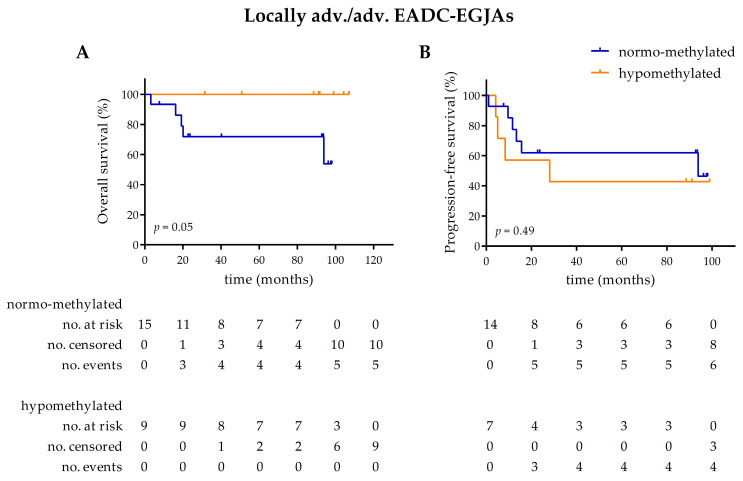
Overall survival and progression-free survival of locally advanced/advanced EADC-EGJA. (**A**) Overall survival. (**B**) Progression-free survival. EADC-EGJA: esophageal adenocarcinoma–esophageal junction adenocarcinoma.

Clinical endpoints (OS and PFS) were analyzed for the locally advanced/advanced EADC-EGJA patients that received surgery, stratified by methylation status. Due to unknown clinical endpoints or lack of surgery, six patients were excluded from the OS, analysis resulting in 24 patients being included (15 normo-methylated and 9 hypomethylated). For the same reason, nine patients were excluded from the PFS analysis, resulting in 21 patients being included (14 normo-methylated and 7 hypomethylated). A borderline difference in OS was observed, with hypomethylated cases showing a longer OS (*p* = 0.05). The median OS of both categories was unreached. We did not find a difference in PFS (*p* = 0.49). The median PFS of the normo-methylated category was 93.83 months and the one of hypomethylated was 28.06 (Figure A1 in Appendix A).

### 3.3. LINE-1 Normalized Methylation Analysis in Cell-Free DNA (cfDNA) of Longitudinal Samples

Twenty-six out of the ninety patients were studied longitudinally by analysis of cfDNA samples isolated from serial blood draws. These patients included 11 NDBE, seven HGD/early EADC-EGJA, and eight locally advanced/advanced EADC-EGJA patients.

All the 11 longitudinally followed NDBE patients had a known clinical history of BE of at least 3 years.

**Figure 4:**
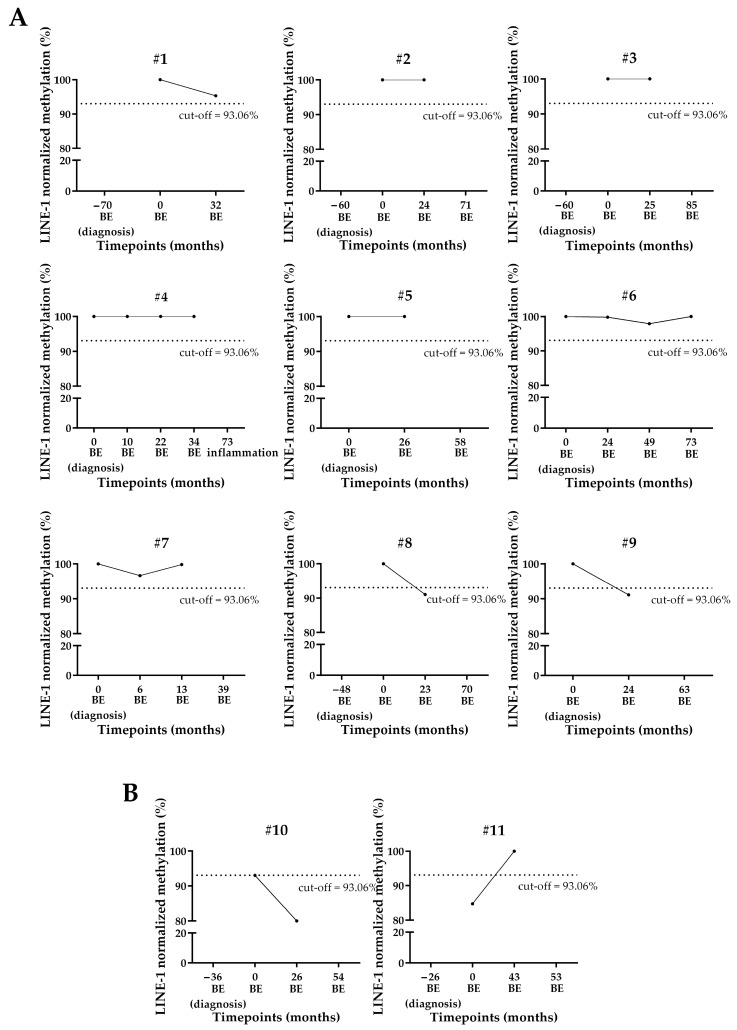
LINE-1 normalized methylation level in longitudinal cfDNA samples of NDBE patients. (**A**) Patients showing normo-methylation at the baseline. (**B**) Patients showing hypomethylation at the baseline. The cut-off of 93.06%, below which the sample was considered as hypomethylated, is shown as a dashed line. LINE-1: long interspersed nuclear element-1; BE: Barrett’s esophagus. Timepoint “0” (baseline) identifies the time of enrollment. Some patients were diagnosed with NDBE prior to enrollment.

Nine out of the eleven NDBE patients were normo-methylated at the first blood draw (timepoint 0): seven of them showed similar methylation levels also in the longitudinally collected plasma samples, whereas two of them (#8 and #9) were hypomethylated at the first follow-up after enrollment (Figure 4A).

Patient #10 showed borderline hypomethylation (93%) at the first blood draw, and the level of methylation decreased further at the follow-up 2 years later (Figure 4B).

The first cfDNA sample of patient #11 was hypomethylated, while the cfDNA collected at the next follow-up, more than three years later, resulted as normo-methylated (Figure 4B).

**Figure 5:**
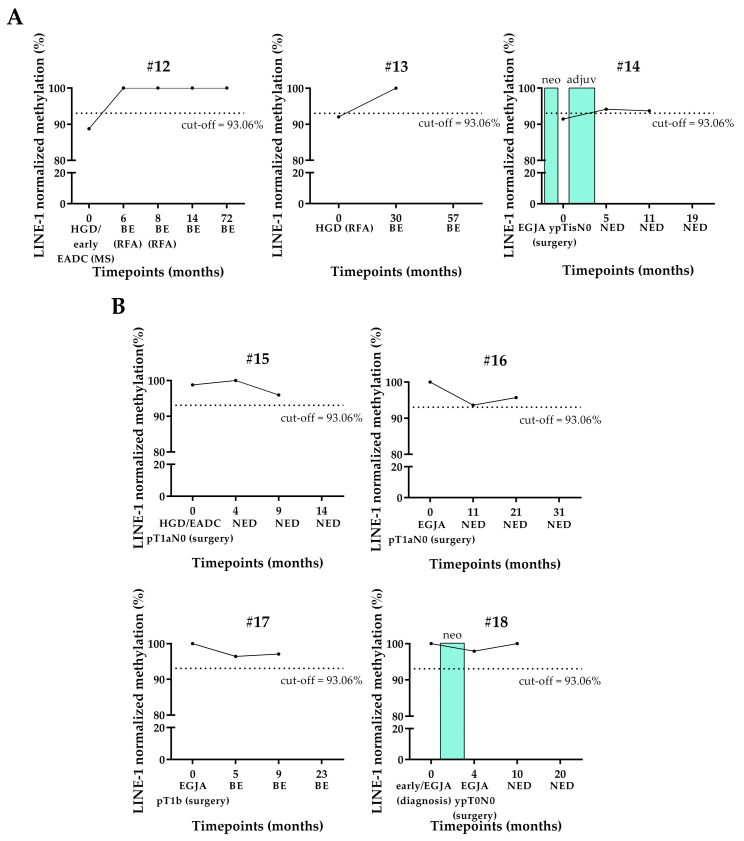
LINE-1 normalized methylation level in longitudinal cfDNA samples of HGD/early EADC-EGJA patients. (**A**) Patients showing normo-methylation at the baseline. (**B**) Patients showing hypomethylation at the baseline. The cut-off of 93.06%, below which the sample was considered as hypomethylated, is shown as a dashed line. LINE-1: long interspersed nuclear element-1; HGD: high-grade dysplasia; EADC: esophageal adenocarcinoma; EGJA: esophageal junction adenocarcinoma; BE: Barrett’s esophagus; MS: mucosectomy; RFA: radiofrequency ablation; NED: no evidence of disease; neo: neoadjuvant therapy; adjuv: adjuvant therapy. Timepoint “0” (baseline) identifies the time of diagnosis or treatment (MS, RFA, or surgery).

All the seven longitudinally studied HGD/early EADC-EGJA patients underwent an endoscopic or surgical treatment for the removal of a lesion, such as RFA, MS, or surgical resection. In three patients (#12, #13, and #14), the cfDNA collected at the time of lesion removal was hypomethylated. Patients #12 and #13, who had BE lesion confirmed by histology taken at the first follow-up after the treatment, had normo-methylated cfDNA at this timepoint (Figure 5A). Patient #14 had the methylation of his cfDNA samples collected at the first and second follow-up after surgery and adjuvant therapy just above the cut-off (Figure 5A). This patient had no evidence of disease (NED) after surgery.

The methylation level of the other four patients (#15, #16, #17, and #18) who were normo-methylated at the surgery or diagnosis remained almost unaltered at their follow-ups several months after surgery. Interestingly, these patients had NED or became BE after surgery (Figure 5B).

**Figure 6:**
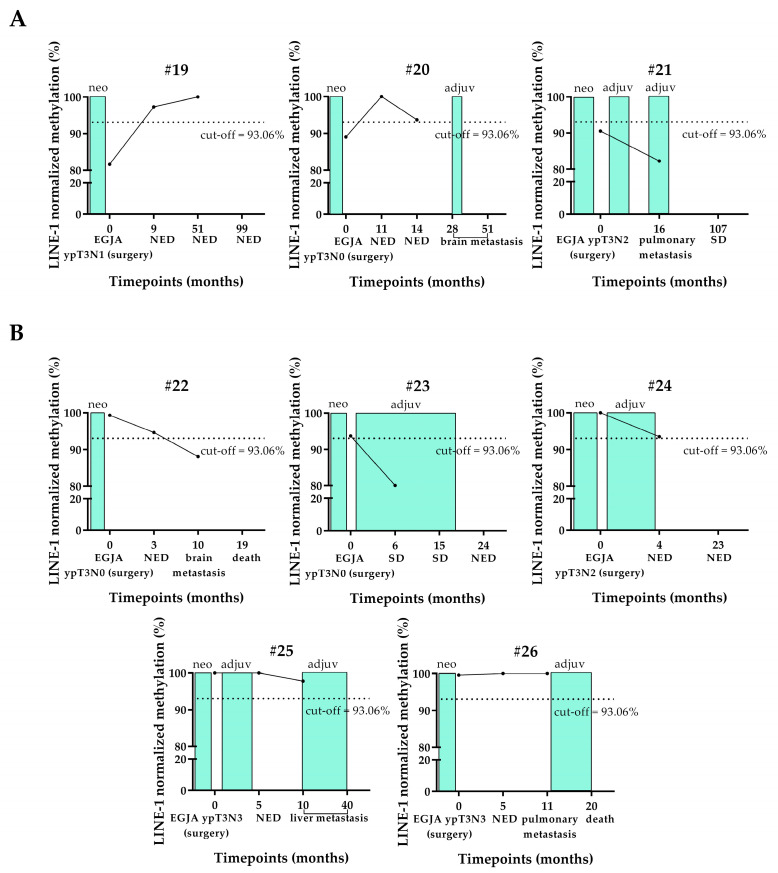
LINE-1 normalized methylation level in longitudinal cfDNA samples of locally advanced/advanced EADC-EGJA patients. (**A**) Patients showing normo-methylation at the baseline. (**B**) Patients showing hypomethylation at the baseline. The cut-off of 93.06%, below which the sample was considered as hypomethylated, is shown as a dashed line. LINE-1: long interspersed nuclear element-1; EGJA: esophageal junction adenocarcinoma; neo: neoadjuvant therapy; adjuv: adjuvant therapy; NED: no evidence of disease; SD: stable disease. Timepoint “0” (baseline) identifies the time of surgery.

The cfDNA sample collected at surgery (baseline) of three out of the eight longitudinally studied locally advanced/advanced EADC-EGJA patients was hypomethylated (Figure 6). Of those three patients showing hypomethylation at the baseline, two (#19 and #20) had a normo-methylated cfDNA sample collected at the first follow-up after esophagectomy, whereas one (#21) still showed hypomethylation at this time-point (Figure 6A).

Patient #19 maintained his cfDNA normo-methylated at the second follow-up, several months after surgery (Figure 6A), when the patient was found with NED.

Patient #20 showed a decreased trend that remained in the range of normo-methylation at the second follow-up 14 months after surgery, in accordance with his NED status. However, more than a year after this point, this patient developed brain metastasis. Unfortunately, the blood draw at this point was not available.

Patient #21 had progression with pulmonary metastasis after surgery. Chemotherapy was administered, leading to stable disease (SD) at the last follow-up (Figure 6A).

Five out of the eight longitudinally studied locally advanced/advanced EADC-EGJA patients were normo-methylated since the time of the resection (Figure 6B).

Two patients (#22 and #23) had a marked hypomethylation of their cfDNA collected at 10 and 6 months after surgery, respectively, in correspondence of brain metastasis (#22) or SD (#23).

Patient #24 had a decrease of methylation level that remained in the range of normo-methylation (borderline) of the cfDNA collected at the first follow-up after surgery, in accordance with his NED status.

For patient #25, we observed, at the timepoint corresponding to liver metastasis, a slightly decreased trend of methylation that was still in the normal range. Unfortunately, an additional blood draw of a successive follow-up, during the adjuvant therapy, was not available to confirm this trend.

Patient #26 had stable normo-methylation despite the occurrence of pulmonary metastasis (Figure 6B).

## 4. Discussion

EADC-EGJA is a very aggressive cancer with poor prognosis, often preceded by BE, its metaplastic precursor. Histologically-defined grading determines the patient’s management [11].

Several unmet clinical needs still exist, such as the identification of NDBE patients who are most at risk of progression and, after cancer diagnosis and treatment, the detection of MRD, which is crucial for the identification of EADC-EGJA patients who are at risk of progression/recurrence post-surgery.

Epigenetic alterations are crucial events involved in the carcinogenesis process of different malignancies [18], and among them, global hypomethylation is linked with genetic instability and pro-carcinogenic mechanisms [20].

This study aimed to investigate the changes in global methylation level during neoplastic progression of EADC-EGJA and to clarify if a global methylation biomarker could be followed to monitor disease behavior. We analyzed the cfDNA samples of a cohort of 90 patients: 30 NDBE, 30 HGD/early EADC-EGJA, and 30 locally advanced/advanced EADC-EGJA patients; additionally, 26 of these patients were longitudinally studied.

To estimate global methylation in cfDNA, the LINE-1 methylation, normalized to gDNA, was used, since this biomarker is a well-known surrogate of this epigenetic event [22].

The data regarding LINE-1 normalized methylation level in the 90 cfDNA samples suggest that global hypomethylation of DNA occurs more frequently in invasive EADC-EGJA compared to dysplasia/early EADC-EGJA and NDBE; however, this epigenetic event can also be present in these latter early conditions.

The tendency to have a more frequent global hypomethylation in the locally advanced/advanced EADC-EGJA group suggests that this alteration could be considered part of the cascade of late dramatic events that drive dysplasia to invasive cancer. In the literature, there are few data about global hypomethylation in EADC-EGJA [39] and, additionally, it is not always clear if data are referring exclusively to EADC-EGJA histology or if ESCC are included [50]. Moreover, there are no data about global hypomethylation in EADC-EGJA pre-neoplastic lesions. More data exist about gastric cancer and its pre-neoplastic lesions. Indeed, three studies have investigated LINE-1 methylation level in tissue samples of gastric cancer, using bisulfite conversion coupled with pyrosequencing [51,52] or digestion with restriction enzymes (COBRA LINE-1) [53]. These studies are concordant in finding a rise of hypomethylation in proximity to neoplastic transformation, while in preneoplastic stages (LGD and gastritis) hypomethylation is very rare [51,52,53].

In two of these three studies, the authors also investigated the correlation of LINE-1 methylation level with age, finding a trend that did not reach significance in the gastric cancer cohort in one study [51] and a significant inverse correlation in male individuals of the gastric cancer cohort in the other [52]. In our study, we did not find a correlation between LINE-1 normalized methylation level and age in NDBE, HGD/early EADC-EGJA, or locally advanced/advanced EADC-EGJA patients, nor when considering the three groups together. A correlation with age was also not found for the healthy controls, which were used to set up the cut-off. Hence, even if they were younger than the patients, this aspect should not be a bias. This result indicates that age is not a possible confounding factor for this biomarker in our population. Moreover, we did not find an association with the main clinicopathological variables, such as length of BE, site of dysplastic lesion/tumor, and stage at the moment of blood draw. However, a clustering of six highly hypomethylated cases was observed. Interestingly, five of them were of males with a locally advanced tumor of at least stage II located at the junction, suggesting that tumors with these clinicopathological characteristics could exhibit a distinctive pronounced hypomethylation.

We found a borderline difference in OS time and no difference in PFS time in the locally advanced/advanced EADC-EGJA population stratified by normalized methylation status. However, we are aware that any conclusion about these clinical endpoints has to be interpreted with caution because the locally advanced/advanced EADC-EGJA group, once dichotomized according to methylation level, was constituted by a small number of patients for each subgroup (especially the hypomethylated one). Hence, the association between LINE-1 normalized methylation level and clinical endpoint should be investigated in a larger cohort of EADC-EGJA patients with a reasonable number of both hypomethylated and normo-methylated tumors.

To our knowledge there are no data in the literature about LINE-1 methylation level and prognosis in EADC-EGJA; indeed, in EC, LINE-1 status has been principally investigated in ESCC, finding frequent hypomethylation associated with poorer survival [50].

Referring again to gastric cancer, the association between LINE-1 hypomethylation and OS is controversial. This discrepancy between studies could be also due to the different ethnicity of studied populations [51,52,54,55]. Hypomethylation has been associated with worse OS in certain tumor types, including colorectal cancer, lung, and ovarian cancer [25,27,36], while it has been associated with a better OS in stage IIIC melanoma patients [56], suggesting that hypomethylation’s effect on OS could be tumor-type-specific.

In our study we analyzed 26 longitudinal cases, following the LINE-1 normalized methylation level in their sequential blood draws collected at each scheduled follow-up.

Results from NDBE patients studied longitudinally showed that the majority of patients maintained LINE-1 normo-methylation for years, confirming the rareness of LINE-1 hypomethylation events in preneoplastic lesions of the esophagus and the stability of a normal level of methylation over time.

Only in four NDBE cases (#8, #9, #10, and #11) did we observe a more complex trend of methylation level that did not perfectly fit with the clinical history. For patient #11, whose cfDNA was hypomethylated at the enrollment and then normo-methylated at the successive blood draws, we hypothesized that hypomethylation may have been a passenger alteration. For the other three patients (#8, #9, and #10), who showed hypomethylation of their last blood draw, it could be intriguing to keep them monitored to check if this phenomenon was a passenger alteration or if it predicted an evolution to dysplasia/adenocarcinoma.

Regarding longitudinal HGD/early EADC-EGJA patients, the majority of them were normo-methylated at surgery and maintained this status during their whole clinical iter, suggesting again that hypomethylation events are quite rare in early lesions. However, for the few cases that showed hypomethylation at the time of surgery, LINE-1 became normo-methylated after the resection according to NED.

The methylation level during the clinical history of six out of eight locally advanced/advanced EADC-EGJA longitudinal cases (#19, #20, #21, #22, #23, and #24), regardless of their methylation status at the baseline surgery point (hypomethylated or normo-methylated), perfectly fit with the clinical status. In particular, normal or altered levels corresponded with NED or disease persistence/progression with metastasis, respectively.

Interestingly, patients #22 and #23 had their baseline point normo-methylated, suggesting that hypomethylated clones may have developed later, at the progression or disease persistence, respectively.

For patient #25, the decreased trend of methylation at 10 months after surgery, even if hypomethylation status was not reached, could explain the occurrence of liver metastasis. However, the lack of other blood draws during the adjuvant therapy to confirm this trend is a limitation for this case.

For patient #26, the methylation level did not fit the clinical status, probably because changes in methylation did not contribute to the carcinogenesis of the tumor, nor to progression with metastasis.

Considering data from the longitudinal studies all together, it emerged that LINE-1 was normo-methylated in the majority of NDBE cases, while in early EADC-EGJA/HGD and locally advanced/advanced EADC-EGJA patients, LINE-1 was more frequently altered and it is a promising marker to monitor the persistence of MRD.

Since patients with advanced adenocarcinoma of the esophagus who undergo curative surgery often develop recurrent disease, finding a biomarker that could predict the postsurgical diagnosis of MRD is of great interest; indeed, MRD detection assays have the potential utility to enable early intervention or adjustments to treatment plans [57,58].

The possibility to use the LINE-1 normalized methylation level in cfDNA as a measure of MRD after surgery has been investigated in a previous study conducted in a Japanese cohort of gastric cancer patients who underwent surgery. The authors found a correlation between post-surgical high concentrations of long-fragment LINE-1 and MRD but they did not find correlation with the methylation level [55]. Besides a difference in ethnicity, the failure of this study to find a correlation with methylation level in post-surgery cfDNA could be explained by the usage of qPCR, which could be influenced by the decrease of tumor cfDNA amount after the resection of tumoral mass. In our opinion, ddPCR, due to the partitioning of the sample into droplets that permit the dilution of the normal cfDNA background, maximizing the chance of rare alterations’ detection, is superior in detecting small differences in methylation level in cfDNA, especially after the removal of tumor mass.

A limitation of this work was that we did not enroll NDBE patients evolving to HGD or up to EADC-EGJA, although it would be appealing to study the trend of this biomarker in longitudinally collected blood draws of those patients. Unfortunately, we could not address this issue because NDBE progressors are very rare [11] and there were none of these patients in our cohort.

An additional limit is that, although for most patients the blood draws at all the key time points were obtained, for a few longitudinal cases some blood draws such as during adjuvant therapy or at the precise occurrence of metastasis are lacking.

Another limit of this study was that we were not able to collect all information for all patients for additional risk factors, like smoking habit, which has been reported to increase the risk of EADC-EGJA in a dose-responsive association, and obesity, which is associated not only with EADC-EGJA and BE but also with the development of dysplasia [6,59].

## 5. Conclusions

In conclusion, our study suggests that global hypomethylation, measured through LINE-1 methylation analysis, occurs just prior to cancer invasiveness and could be a promising liquid biopsy biomarker to monitor the presence of MRD in HGD or locally advanced/advanced EADC-EGJA patients who undergo endoscopic or surgical resection. Future studies with a larger number of EADC-EGJA patients longitudinally followed via liquid biopsy samples would help to confirm our findings on this crucial and currently unmet clinical need.

## Figures and Tables

**Table 1:** Clinicopathological characteristics of NDBE, HGD/early EADC-EGJA, and locally advanced/advanced EADC-EGJA patients.

Patients	NDBE	HGD/Early EADC-EGJA	Locally adv./ adv. EADC-EGJA
	N (%)	N (%)	N (%)
	30 (33.3%)	30 (33.3%)	30 (33.3%)
**Age**			
Median (min; max)	54.5 (21; 75)	68 (36; 86)	66 (41; 87)
IQR	14.2	10.2	18
**Gender**			
Male	25 (83.3%)	27 (90%)	26 (86.7%)
Female	5 (16.7%)	3 (10%)	4 (13.3%)
**Length of BE lesion**			
Short (<3 cm)	5 (16.67%)	/	/
Long (≥3 cm)	25 (83.33%)	/	/
**Tumor site of lesion/** **tumor**			
Esophagus	30 (100%)	26 (86.67%)	6 (20%)
Esophageal junction	0 (0%)	4 (13.33%)	24 (80%)
**cStage**			
0	/	21 (70%)	0 (0%)
I	/	9 (30%)	0 (0%)
III	/	0 (0%)	22 (73.3%)
IVA	/	0 (0%)	3 (10%)
IVB	/	0 (0%)	2 (6.7%)
Unknown	/	0 (0%)	3 (10%)
**pStage**			
0	/	2 (18.18%) *	0 (0%)
I	/	9 (81.82%) *	3 (10%)
II-IIIB	/	0 (0%)	16 (53.3%)
IVA	/	0 (0%)	5 (16.7%)
Unknown (no surgery)	/	0 (0%)	6 (20%)
**Neoadjuvant treatment**			
Yes	/	2 (6.67%)	29 (96.7%)
No	/	28 (93.33%)	1 (3.3%)
**Adjuvant treatment**			
Yes	/	/	12 (40%)
No	/	/	18 (60%)

NDBE: non-dysplastic Barrett’s esophagus; HGD: high-grade dysplasia; EADC: esophageal adenocarcinoma; EGJA: esophagogastric junction adenocarcinoma; IQR: interquartile range; BE: Barrett’s esophagus; cStage: clinical tumor stage; pStage: pathological tumor stage; ypStage: post-neoadjuvant therapy pathological tumor stage. * refers only to early EADC-EGJA patients who underwent surgery.

## Data Availability

The original contributions presented in this study are included in the article/Supplementary Materials. Further inquiries can be directed to the corresponding author.

## Supplementary Materials

The following supporting information can be downloaded at: https://www.mdpi.com/article/10.3390/cancers17162668/s1, Table S1: Raw methylation of cfDNA and gDNA in the different patients’ groups and controls.

## Abbreviations

The following abbreviations are used in this manuscript:

AJCC	American Joint Committee on Cancer
BE	Barrett’s esophagus
cfDNA	cell-free DNA
ddPCR	droplet digital PCR
EADC	esophageal adenocarcinoma
EC	esophageal cancer
EGJA	esophagogastric junction adenocarcinoma
ESCC	squamous cell carcinoma
gDNA	germline DNA
GERD	gastro-esophageal reflux disease
HGD	high-grade dysplasia
IQR	interquartile range
LGD	low-grade dysplasia
LINE-1	long-interspersed nuclear element-1
MRD	Minimal residual disease
MS	mucosectomy
MSRE-ddPCR	Methylation-Sensitive Restriction Enzyme and droplet digital PCR
NDBE	non-dysplastic Barrett’s esophagus
NED	no evidence of disease
OS	overall survival
PFS	progression-free survival
RFA	radiofrequency ablation
SD	stable disease
