# Supplementary material for: Global Hypomethylation as Minimal Residual Disease (MRD) Biomarker in Esophageal and Esophagogastric Junction Adenocarcinoma
Source: Cancers (Basel). 2025 Aug 15;17(16):2668. doi: 10.3390/cancers17162668 (PMC12384112; doi:10.3390/cancers17162668)
Supplement: Supplementary file 1 [file cancers-17-02668-s001.zip › cancers-3641896-supplementary.pdf]

Table S1: Raw methylation of cfDNA and gDNA in the different patients' groups and controls

locally advanced/advanced EADC-EGJA						
**n°copies of digested cfDNA/µl**	**n°copies of undigested cfDNA/µl**	**n°copies of digested gDNA/µl**	**n°copies of undigested gDNA/µl**	**raw methylation of cfDNA (ratio between n° copies of digested/undigested cfDNA)**	**raw methylation of gDNA (ratio between n° copies of digested/undigested gDNA)**	**LINE-1 normalized methylation (ratio between raw methylation of cfDNA and raw methylation of gDNA)**
145.50	158.00	120.00	141.00	0.92	0.85	108.20
131.00	179.50	226.00	251.00	0.73	0.90	81.05
139.50	156.00	87.30	104.00	0.89	0.84	106.53
195.00	214.00	219.50	255.50	0.91	0.86	106.07
154.50	167.00	172.50	187.00	0.93	0.92	100.29
84.50	101.50	191.00	193.50	0.83	0.99	84.34
119.50	152.50	76.00	87.90	0.78	0.86	90.63
33.60	43.30	136.00	164.00	0.78	0.83	93.57
53.85	65.55	99.20	123.00	0.82	0.81	101.86
97.00	131.50	114.50	123.00	0.74	0.93	79.24
220.50	275.50	92.00	115.50	0.80	0.80	100.48
146.00	161.50	167.00	191.00	0.90	0.87	103.39
48.10	61.35	162.00	212.50	0.78	0.76	102.84
90.50	107.50	119.00	128.00	0.84	0.93	90.55
120.50	138.50	139.00	167.50	0.87	0.83	104.84

102.50	128.00	303.00	348.00	0.80	0.87	91.97
119.50	129.00	97.00	111.50	0.93	0.87	106.48
123.00	132.50	193.00	207.50	0.93	0.93	99.80
148.50	217.00	124.50	148.50	0.68	0.84	81.63
272.00	289.00	345.50	375.50	0.94	0.92	102.29
227.50	245.50	438.00	469.50	0.93	0.93	99.33
93.50	108.00	219.50	252.50	0.87	0.87	99.59
136.50	175.50	176.50	202.00	0.78	0.87	89.01
96.45	97.00	75.00	77.00	0.99	0.97	102.08
55.70	74.30	528.50	621.50	0.75	0.85	88.16
127.00	160.50	260.50	308.50	0.79	0.84	93.71
240.50	265.50	172.00	221.00	0.91	0.78	116.39
133.50	148.50	346.00	396.50	0.90	0.87	103.02
321.00	402.00	313.50	308.00	0.80	1.00	79.85
193.50	230.00	150.00	180.00	0.84	0.83	100.96
**HGD/early EADC-EGJA**						
262.50	259.50	116.00	126.00	1.00	0.92	108.62
49.15	55.40	123.50	142.00	0.89	0.87	102.01
213.00	235.50	109.50	116.50	0.90	0.94	96.23
152.50	161.00	124.50	128.50	0.95	0.97	97.76
38.70	47.80	104.30	119.00	0.81	0.88	92.37
88.25	106.15	94.75	107.90	0.83	0.88	94.68
46.35	58.70	41.50	51.25	0.79	0.81	97.51
64.80	74.40	93.35	106.00	0.87	0.88	98.90
80.05	82.50	118.00	132.50	0.97	0.89	108.95

131.00	147.50	145.00	175.00	0.89	0.83	107.19
120.00	157.00	211.00	245.00	0.76	0.86	88.75
59.00	78.00	182.00	221.50	0.76	0.82	92.06
241.50	239.50	262.00	301.00	1.00	0.87	114.89
142.00	156.00	153.00	199.00	1.00	0.77	130.07
189.50	217.00	112.50	135.00	1.00	0.83	120.00
78.50	83.50	106.00	123.50	1.00	0.86	116.51
73.50	76.00	246.50	269.00	0.97	0.92	105.54
140.50	160.50	330.00	377.00	0.88	0.88	100.01
231.00	252.50	274.50	324.50	0.91	0.85	108.15
217.50	250.00	255.50	280.00	0.87	0.91	95.34
224.50	254.00	319.00	368.00	0.88	0.87	101.96
287.00	292.00	153.00	158.00	0.98	0.97	101.50
258.00	327.50	362.00	454.00	0.79	0.80	98.80
208.50	215.00	60.65	65.75	0.97	0.92	105.13
156.50	187.50	125.50	137.50	0.83	0.91	91.45
118.50	122.00	234.00	242.00	0.97	0.97	100.45
202.00	232.00	253.00	284.50	0.87	0.89	97.91
203.50	257.00	284.50	311.50	0.79	0.91	86.70
195.50	229.00	313.50	331.00	0.85	0.95	90.14
124.00	157.00	108.00	139.50	0.79	0.77	102.02
**non-dysplastic Barrett's esophagus (NDBE)**						
98.00	102.50	76.85	87.30	0.96	0.88	108.61
35.05	41.95	151.00	151.00	0.84	1.00	83.55
179.50	210.00	89.30	109.85	0.85	0.81	105.15

33.80	38.50	145.50	158.50	0.88	0.92	95.64
162.00	162.50	110.50	120.00	1.00	0.92	108.26
39.95	48.05	100.50	120.50	0.83	0.83	99.69
58.30	69.25	139.50	154.50	0.84	0.90	93.24
298.50	318.00	219.50	269.00	0.94	0.82	115.04
92.50	109.00	272.50	312.00	0.85	0.87	97.16
156.50	167.50	194.00	233.00	0.93	0.83	112.22
45.50	64.50	77.90	105.50	0.71	0.74	95.54
128.00	142.50	211.00	230.00	0.90	0.92	97.91
50.30	56.10	241.50	288.50	0.90	0.84	107.11
75.50	84.00	179.00	247.00	0.90	0.72	124.03
89.50	110.00	257.50	299.50	0.81	0.86	94.63
238.50	289.50	43.40	53.00	0.82	0.82	100.61
121.00	148.00	212.50	259.50	0.82	0.82	99.84
112.00	107.00	180.50	187.00	1.05	0.97	108.44
163.50	171.50	272.50	309.50	0.95	0.88	108.28
220.00	224.50	229.50	277.50	0.98	0.83	118.49
114.50	129.50	276.00	311.00	0.88	0.89	99.63
211.50	222.00	402.50	435.00	0.95	0.93	102.96
151.50	177.00	100.00	106.00	0.86	0.94	90.73
182.50	199.50	239.50	282.50	0.91	0.85	107.90
133.00	155.50	233.00	279.50	0.86	0.83	102.60
286.50	306.50	249.00	232.00	0.93	1.00	93.47
311.00	329.50	315.00	342.00	0.94	0.92	102.48
104.00	117.00	247.50	253.00	0.89	0.98	90.86
224.00	261.00	382.50	418.50	0.86	0.91	93.90

289.00	339.00	262.50	294.50	0.85	0.89	95.64
**Controls**						
60.50	69.60	197.50	245.00	0.87	0.81	107.83
48.20	57.55	433.50	469.00	0.84	0.92	90.61
33.55	41.00	216.00	252.00	0.82	0.86	95.47
90.50	97.00	239.00	248.00	0.93	0.96	96.81
95.50	113.50	248.50	324.00	0.84	0.77	109.70
54.70	60.00	70.00	122.00	0.91	0.57	158.89
98.50	107.50	200.00	224.00	0.92	0.89	102.62
111.50	128.50	198.00	229.50	0.87	0.86	100.57
105.00	108.00	171.50	217.00	0.97	0.79	123.02
137.00	164.50	222.00	284.00	0.83	0.78	106.54
130.00	170.50	152.50	184.00	0.76	0.83	92.00
181.50	224.50	202.50	265.00	0.81	0.76	105.80
97.00	106.50	301.00	358.50	0.91	0.84	108.48
98.50	100.50	168.50	188.00	0.98	0.90	109.35
129.00	140.00	123.50	151.00	0.92	0.82	112.66
117.00	144.50	339.50	422.50	0.81	0.80	100.76
63.75	55.00	243.00	272.50	1.00	0.89	112.14
102.50	115.00	90.50	119.00	0.89	0.76	117.20
96.50	112.00	52.20	74.40	0.86	0.70	122.80
95.50	101.00	283.00	295.00	0.95	0.96	98.56
